# Fluorescence characterisation and visual ecology of pseudocheilinid wrasses

**DOI:** 10.1186/s12983-016-0145-1

**Published:** 2016-03-15

**Authors:** Tobias Gerlach, Jennifer Theobald, Nathan S. Hart, Shaun P. Collin, Nico K. Michiels

**Affiliations:** Animal Evolutionary Ecology group, Faculty of Sciences, University of Tübingen, Tübingen, Germany; School of Animal Biology and The Oceans Institute, The University of Western Australia, Perth, Australia

**Keywords:** Red fluorescence, Photoluminescence, Microspectrophotometry, Colour vision, Labridae

## Abstract

**Background:**

Wrasses represent the second largest family of marine fishes and display a high diversity of complex colours linked to ecological functions. Recently, red autofluorescent body colouration has been reported in some of these fishes. However, little is known about the distribution of such fluorescent body patterns in wrasses or the animals’ ability to perceive such colours.

**Results:**

Against this background, we (1) investigated long-wavelength emission autofluorescence in thirteen species of pseudocheilinid wrasses and (2) characterised the spectral absorbance of visual pigments in one of the examined species, the fairy wrasse *Cirrhilabrus solorensis*. Spectrophotometric analysis revealed that fluorescent body colouration is widespread and diverse within this clade, with considerable variation in both fluorescent pattern and maximum emission wavelength between species. Characterisation of visual pigments in retinal photoreceptors showed a single class of rod and three spectrally distinct cone photoreceptors, suggesting possible trichromacy.

**Conclusion:**

Combining the emission characteristics of fluorescence body colouration and the spectral sensitivity data of retinal cells suggests that the visual system of *C. solorensis* is sensitive to pseudocheilinid fluorescence.

**Electronic supplementary material:**

The online version of this article (doi:10.1186/s12983-016-0145-1) contains supplementary material, which is available to authorized users.

## Background

Wrasses (Labridae) represent the second largest marine fish family, containing more than 600 species within 82 genera [1]. They are one of the most morphologically and ecologically diverse clades of marine teleosts [2–4] and are a dominant group in reef fish communities [5]. Wrasses display a staggering diversity of complex colours virtually unique to this family [6, 7]. These colours have been linked to important ecological functions such as camouflage and aggressive mimicry [8], interspecific signalling [9] as well as courtship and male competition [10–12]. Yet, the range of colours that can bear such ecological functions is limited by two factors: the ability to generate colours under water and the visual capabilities of the fish.

Many wrasses live in the spectrally restricted part of the ocean – the ‘stenospectral zone’ [13] – where reflective colours do not appear as they do at the surface [14]. That is because with increasing depth, the long-wavelength (>600 nm) part of downwelling sunlight is quickly absorbed by sea water, which is most transparent to blue light of wavelengths at around 480 nm [15–17]. This lack of long-wavelength sunlight below about 10 to 20 m depth inhibits red and orange reflective colouration, which consequently appears grey or black under these conditions [18]. In contrast, recently described red fluorescence in a wide variety of reef fishes – including wrasses – [19, 20] constitutes a fundamentally different mechanism, where fluorescent structures absorb ambient short-wavelength blue light and re-emit photons at longer wavelengths. This process can enable the display of red colour even at depths devoid of red sunlight. Hence, fluorescence can generate conspicuous colour contrasts, particularly in the near monochromatic light environment prevalent in most parts of the ocean [18]. Yet for now, there is only limited data on both the perception and function of red fluorescence in fishes.

Research on the visual capabilities of reef fishes has advanced greatly with the use of microspectrophotometry (MSP) to measure the spectral absorbance of individual photoreceptors. This has led to a growing body of data with photoreceptor sensitivities of more than 80 reef fish species characterised to date [21–25]. However, despite the high number of species and the ecological importance of labrids, only few studies have analysed photoreceptor sensitivities in wrasses [7, 26] and therefore the visual capabilities of this family is mostly unknown.

This study investigates long-wavelength (>640 nm) fluorescence in 13 species of pseudocheilinid wrasses. Pseudocheilines are a monophyletic clade [1] of diurnal zooplanktivores (but see also [27]) and inhabit the base of tropical reefs at depths of 20 to 50 m, some venturing as deep as 200 m [4, 28–32], well within the stenospectral zone devoid of red sunlight. When first describing fluorescence in reef fishes, Michiels et al. [19] noted two autofluorescent pseudocheilinid species, *Pseudocheilinus evanidus* [33] and *Paracheilinus octotaenia* [34]. Since then, fluorescence has been observed in more than 180 reef fish taxa, often in complex, clade-dependent patterns [20] and has been suggested to play a role in intraspecific communication [19]. Indeed, recent behavioural experiments have shown that the pseudocheilinid fairy wrasse *Cirrhilabrus solorensis* [35] can perceive its own red fluorescent body colouration and that this fluorescent colour affects agonistic male-male interactions [36].

This work aims to characterise the visual system of the pseudocheilinid wrasse *C. solorensis* by reporting microspectrophotometric measurements of the spectral absorbances of each of the retinal photoreceptor types as well as the spectral transmittance data on the ocular media. Linking this information on the visual capabilities of this species with the deep red fluorescence featured in Pseudocheilines, enables a more complete analysis of colour perception in labrids in general and the role of long-wavelength fluorescence in particular.

## Results

### Fluorescence characterisation

**Fig. 1:**
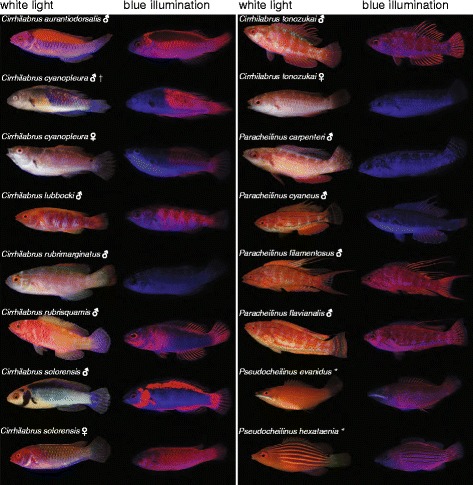
Fluorescent patterns across species. All species investigated photographed under broad-spectrum white light and monochromatic blue illumination in combination with optical long pass filter (Lee filter 105 “orange”, see methods for details). Relative body sizes are not to scale, but given in Table 2. ♂ = terminal phase/male; ♀ = initial phase/female; * = unsexed; † = animal died after fluorescence spectrometry

Fluorescent body patterns varied greatly across species and, in the few cases examined, between sexes. Fluorescence was observed in the fins – especially in the dorsal and caudal fin rays – and in many cases formed blotches near the operculum or stripes in the dorsolateral region of the body. To illustrate different fluorescent patterns among species, both blue illuminated fluorescence images and broad-spectrum white light images are displayed in Fig. 1.

**Fig. 2:**
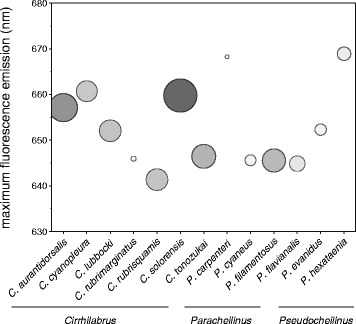
Wavelength of maximum fluorescent emission across species. Size of circles depicts relative fluorescent brightness

Peak fluorescence emission in all 13 species examined ranged from 641 nm in *Cirrhilabrus rubrisquamis* [37] to 669 nm in *Paracheilinus carpenteri* [38] and *Pseudocheilinus hexataenia* [39]. Moreover, fluorescent brightness differed strongly across species. Fluorescence is characterised by wavelength of maximum emission and fluorescent brightness in Fig. 2.

### Microspectrophotometry

**Fig. 3:**
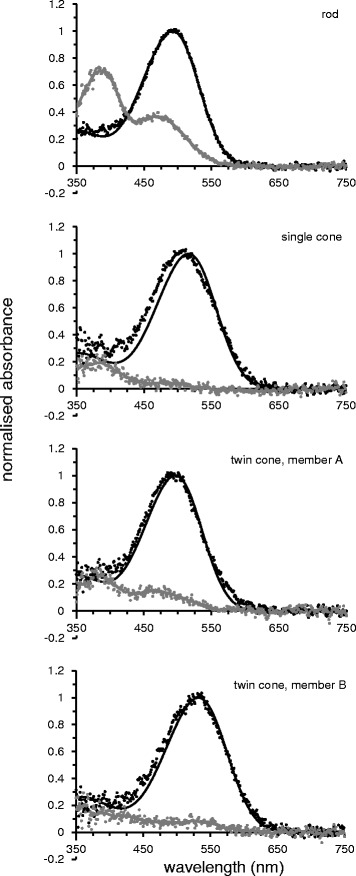
Photoreceptor absorbance spectra. Normalised and averaged mean pre-bleach (*black dots*) as well as post-bleach (*grey dots*) absorbance spectra and their respective best-fitted rhodopsin-templates (*black lines*) of visual pigments in *Cirrhilabrus solorensis*. Deviations of measured single cone absorbance from its rhodopsin template suggest that single cones express more than one visual pigment (see results for details)

**Fig. 4:**
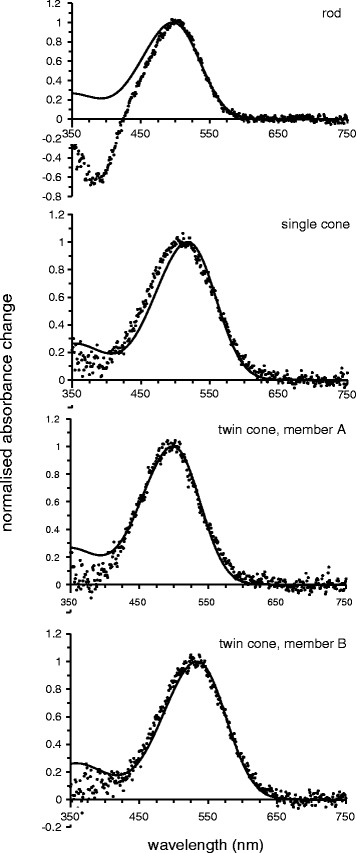
Photoreceptor difference spectra. Normalised mean difference spectra (*black dots*) and their respective best-fitted rhodopsin templates (*black lines*) of visual pigments in *Cirrhilabrus solorensis*

Microspectrophotometric data for all photoreceptor classes found in the fairy wrasse *Cirrhilabrus solorensis* are summarised in Table 1 and displayed in Figs. 3 and 4. All visual pigment absorbance spectra are considered to represent vitamin A_1_-based visual pigments (i.e. rhodopsins), according to the fit of the data to A_1_ visual pigment templates [40]. The retina of *C. solorensis* contains at least four spectrally distinct visual pigments in three classes of photoreceptors: one type of rod containing a visual pigment with a mean wavelength of maximum absorbance (*λ*_max_) of the pre-bleach spectra at 492.3 ± 1.0 nm; one type of single cone with *λ*_max_ of 514.1 ± 6.6 nm and one type of twin cone.

**Table 1:** Characteristics of photoreceptor classes in the retina of *C. solorensis; n* = 10 receptor cells per photoreceptor type; values are shown ± one standard deviation

	Rod	Single cone	Twin cone, member A	Twin cone, member B
Mean *λ*_max_ of pre-bleach absorbance spectra (nm)	492.3 ± 1.0 nm	514.1 ± 6.6 nm	497.7 ± 6.3 nm	532.4 ± 3.0 nm
Mean absorbance at *λ*_max_ of pre-bleach spectra	0.033 ± 0.007	0.030 ± 0.012	0.024 ± 0.006	0.025 ± 0.004
Mean *λ*_max_ of difference spectrum (nm)	496.6 ± 2.7 nm	516.2 ± 6.8 nm	499.2 ± 5.4 nm	533.7 ± 2.7 nm
Absorbance at *λ*_max_ of mean difference spectrum	0.023 ± 0.006	0.029 ± 0.012	0.021 ± 0.006	0.021 ± 0.005
Approximate dimensions of receptor outer segment	2 × 14 μm	1.5 × 3 μm	1.5 × 3 μm	1.5 × 3 μm

The twin cones possess different visual pigments in their respective cone members with *λ*_max_ at 497.7 ± 6.3 nm in one member and 532.3 ± 3.0 nm in the other member. Inspection of individual scans from single cone outer segments and also the mean spectrum constructed from all acceptable single cone records suggest the possibility that single cones express more than one visual pigment: the absorbance spectrum deviates markedly from the template on the short-wavelength limb, i.e. has a greater spectral bandwidth, and the running average of the peak of the spectrum (507 nm) is different to the estimate of *λ*_max_ obtained by fitting a regression line to the long wavelength limb (516 nm). Based on the data for rods and twin cones, we assume that all pigments are A_1_-based. We modelled the fit to the mean spectra of a combination of two A_1_-based visual pigments (different opsins) following the methods described elsewhere [41], which gave predicted *λ*_max_ values of 485 nm (41 %) and 527 nm (59 %) for the mean pre-bleach spectrum, and 489 nm (44 %) and 530 nm (56 %) for the mean difference spectrum. Both of the predicted *λ*_max_ values are very close to the estimated *λ*_max_ values of the pigments contained in the two members of the twin cone. The photoreceptor classes can be differentiated morphologically: rods are characterised by their long, cylindrical outer segments, while single cones feature shorter, conical outer segments. Twin cones comprise two members that resemble single cones in size and shape but have closely opposed inner and outer segments. For each receptor class, approximate sizes of the outer segments are given in Table 1.

### Ocular media transmittance

**Fig. 5:**
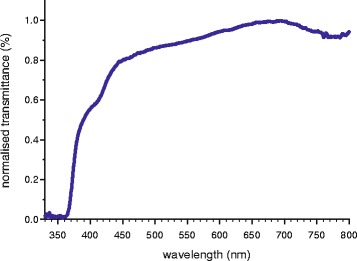
Ocular media transmittance. Normalised mean (*n* = 6 measurements) transmittance spectrum of the ocular media of *Cirrhilabrus solorensis*, measured along the optical axis. The wavelength at 0.5 transmittance (T_0.5_) is 389 nm

The spectral transmittance of ocular media taken from two whole *C. solorensis* eyes is shown in Fig. 5. The wavelength of 0.5 transmittance (*T*_0.5_) is 389 nm and no wavelengths shorter than approximately 360 nm can reach the retina.

## Discussion

The Pseudocheilines investigated here display a high diversity of deep red fluorescence: our data show variation in fluorescent peak emission and fluorescent brightness among species. In *Cirrhilabrus cyanopleura* [42], *C. solorensis* and *C. tonozukai* [43], both terminal-phase males and initial-phase females were analysed and distinct differences in relative fluorescence intensity and pattern, but not peak emission wavelength, were observed between sexes. These differences across species and sexes suggest that red fluorescence can potentially serve species recognition as well as mate choice and corroborate a potential signalling function [19, 20, 36, 44, 45].

Our microspectrophotometric data show that the fairy wrasse *C. solorensis* possesses three spectrally distinct cone visual pigments in one class of single cone and one class of twin cone. Although abundant in most teleost fish, birds, reptiles and marsupials, the exact function of twin- or double cones is not fully understood [24]. While twin cones have long been associated with achromatic perception tasks such as luminance and polarisation detection [46–48], recent behavioural experiments have shown that one species reef fish can use twin cones for colour discrimination [49, 50]. It has thus been suggested that some fishes with one type of visual pigment in single cones and two different pigments in each member of twin cones are effectively trichromatic [49]. The photoreceptor visual pigment *λ*_max_ values of absorbance values of *C. solorensis* presented here are similar to existing data on other reef fish families [26] and generally match the blue dominated light environment, but lack a distinct UV cone receptor found in some shallow water species [26, 51]. This indicates that the visual system of fairy wrasses is well adapted to their stenospectral habitat, as predicted by the *sensitivity hypothesis* [21, 52].

The range of wavelengths an animal can perceive is not only dependent on the sensitivity of its visual pigments, but also on the wavelengths that can reach the retina [53] and so any interpretation of microspectrophotometric measurements must regard the ocular media spectral transmittance. Our data on whole eye samples in *C. solorensis* show a relatively high spectral transmittance across most of the spectrum with a short wavelength cut-off at approximately 360 nm. These results are consistent with previous work on ocular media in labrids, specifically data on *Cirrhilabrus punctatus* [54, 55].

**Fig. 6:**
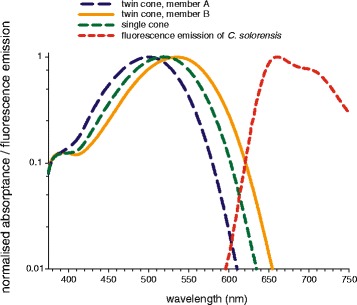
Linking fluorescence with spectral sensitivities. Fluorescence emission in *Cirrhilabrus solorensis* combined with spectral sensitivity of cone photoreceptors, corrected for ocular media transmittance

Is the potentially trichromatic visual system of pseudocheilinid wrasses sensitive to their own long-wavelength fluorescence? When correcting the cone absorptance of *C. solorensis* for ocular media transmittance and combining the resulting spectral sensitivities with the fluorescence emission of the same species, there is a partial overlap at the long-wavelength sensitive twin cone member B (Fig. 6). While it is hard to predict how such an overlap is processed by the visual system, our physiological data indicate that the visual system of *C. solorensis* is sensitive to at least part of the fluorescence emitted by the Pseudocheilines characterised here. This notion is supported by our recent behavioural study in *C. solorensis*, where red fluorescent body colouration has been shown to affect male agonistic reactions [36]. Microspectrophotometric measurements on the red fluorescent goby *Eviota atriventris* [56] indicate that this species, too, is capable of seeing its own fluorescence [19].

It has been argued that *λ*_max_ values of photoreceptors are often shorter than expected due to physiological constraints of long-wavelength sensitive pigments, which are affected by thermal noise [17, 57]. However, it is important to note that colour vision is not the result of how much a certain receptor is stimulated in isolation, but how much it is stimulated in relation to other receptor types [58]. By implication, even relatively weak long-wavelength absorbances can be sufficient to perceive a signal.

## Conclusion

Our findings illustrate the presence and remarkable diversity of long-wavelength fluorescence in Pseudocheilines and provide descriptive data on their visual system, for the first time showing potential trichromacy in labrids. These physiological measurements lay the foundation for future experiments on the potential functions of red fluorescence in reef fish.

## Methods

Fluorescence characterisation of 13 species of Pseudocheilines (Table 2) took place at the University of Tübingen, Germany, approved by local state authority under permit no. ZO 1/12. Microspectrophotometric (MSP) and spectrophotometric (ocular media transmittance) measurements of *Cirrhilabrus solorensis* were carried out at the University of Western Australia, following procedures approved by the UWA Animal Ethics Committee (RA/3/100/1220). All animals were obtained from ornamental fish traders (von Wussow Importe, Pinneberg, Germany and Oceanreef, Wangara, Australia).

**Table 2:** List of species, sex and standard length (*L*_S_) of fish examined

Species	Sex	*L*_S_ (mm)
*Cirrhilabrus aurantidorsalis*	male	62.0
*Cirrhilabrus cyanopleura*	male	81.9
*Cirrhilabrus cyanopleura*	female	52.9
*Cirrhilabrus lubbocki*	male	45.8
*Cirrhilabrus rubrimarginatus*	male	55.5
*Cirrhilabrus rubrisquamis*	male	62.3
*Cirrhilabrus solorensis*	male	67.6
*Cirrhilabrus solorensis*	female	84.1
*Cirrhilabrus tonozukai*	male	47.0
*Cirrhilabrus tonozukai*	female	36.3
*Paracheilinus carpenteri*	male	53.6
*Paracheilinus cyaneus*	male	50.3
*Paracheilinus filamentosus*	male	61.0
*Paracheilinus flavianalis*	male	66.5
*Pseudocheilinus evanidus*	unsexed	27.4
*Pseudocheilinus hexataenia*	unsexed	32.5

- Fluorescence characterisation
  For fluorescence characterisation, each fish was first sedated using cooled water and then placed onto its right flank in a water-filled petri dish lined with non-reflective black cloth. Spectrometric measurements were taken with a cooled, low-noise spectrophotometer (QE65000, Ocean Optics, Florida, USA), a bifurcated fibre optic cable (Ocean Optics QR400-7-VIS-BX) with a waterproof probe and SpectraSuite software (Ocean Optics, v. 2.0.132). A green light laser excitation source (CPS532 Thorlabs, New Jersey, USA) was combined with a short pass filter to clean up the excitation signal (BrightLine HC 533/SP, AHF Analysetechnik, Tübingen, Germany) at the incoming arm and a long-pass filter (EdgeBasic 532R-25, Semrock, New York, USA) in the outgoing arm that leads the emitted light from the sample into the spectrometer. Each measurement was taken with the probe tip submerged and held at an angle of 45° relative to the surface of the fish (see also [13]). Every animal was measured repeatedly on several predefined locations: the eye, dorsal part of the head, the operculum, both ventral and dorsal parts of the lateral body as well as each fin. Within all individuals, peak fluorescent emission was highly consistent across these body parts. Hence, for each species investigated, a single summarising measurement of peak fluorescence emission (*λ*_max_) and maximum fluorescent brightness was obtained [13].
  Following spectrometry, each animal was transferred into a small, custom-built photo chamber featuring a scale bar. The fish was then photographed under broad spectrum white light and monochromatic blue light from two 450 nm LED torches (mini compact LCD, Hartenberger, Köln, Germany) each in combination with a short pass filter (Thorlabs FD2C) for a sharper excitation cut-off. A digital still camera (Canon EOS 7D) and an EF-S 60 mm f/2.8 macro lens was used in combination with an optical long-pass filter (LEE filter no. 105, Hampshire, UK) for fluorescence images. The latter attenuated the excitation light (<550 nm) and enhanced the visibility of long wavelength fluorescence [36]. Pictures were used to graphically illustrate different fluorescent patterns and to assess standard length (*L*_S_, i.e. the distance from the snout to the caudal peduncle) in ImageJ v. 1.45 s [59].
- Microspectrophotometry of visual pigments
  Five male *Cirrhilabrus solorensis* (*L*_S_ = 4.5–6.1 cm) were used for MSP analysis. Animals were dark-adapted for 1 h prior to being euthanised with an overdose of tricaine methanesulphonate salt (MS222). Retinal tissue samples were collected as described in detail elsewhere [23, 24]. In short, both eyes of each specimen were removed and dissected under infrared (IR) illumination using an IR image converter mounted on a dissecting microscope in order to avoid bleaching of photopigments. One eye was immersed in Hickman’s teleost ringer (420 mOsmol kg^−1^) for immediate dissection and MSP analysis, while the other eye was stored in a light-tight container at 4 °C for use at the following day. The eye was hemisected and both the lens and the vitreous humour removed. The retina was then extracted, dissected into several small pieces (approximately 1–3 mm^2^) and each piece transferred into a drop of teleost ringer solution containing 8 % dextran (MW 282,000; Sigma D-7265) sitting on a glass coverslip. A second coverslip was placed on top of the retinal sample and the edges sealed with nail varnish. Individual samples thus prepared were stored at 4 °C (see [23]) and analysed within the same day.
  Absorbance spectra (330–800 nm) of visual pigments within photoreceptor outer segments were measured using a computer-controlled, single-beam, wavelength-scanning microspectrophotometer (for details, see [60]). For each photoreceptor cell examined, a sample and baseline scan was made from cellular and tissue-free regions of the preparation, respectively [60, 61]. The baseline transmittance was subtracted from the sample resulting in a pre-bleach spectrum. In order to ensure that the measured spectrum originated from a photolabile visual pigment, each outer segment was then bleached with full spectrum (‘white’) light for 2 min and subsequent sample and baseline scans were used to create a post-bleach spectrum. This post-bleach spectrum was subtracted from the pre-bleach spectrum to calculate a bleaching difference spectrum for each photoreceptor outer segment [23, 61]. Individual absorbance spectra were normalised and then analysed as described in [60] and [23]. Briefly, peak and long-wavelength offset absorbances were determined by fitting a variable-point unweighted running average to the data [62]. Following the methods of Govardovskii [40], a regression line was then fitted to the normalised absorbance spectrum between 30 and 70 % of the normalised maximum on the long-wavelength limb to predict the wavelength of maximum absorbance (*λ*_max_). Only spectral measurements that satisfied established selection criteria (e.g. free from distortion and confirmed as photolabile, see [62]) were included in the final analysis. Ten such difference spectra were averaged from separate cell outer segments to calculate mean *λ*_max_ values of each photoreceptor type. For display purposes, averaged spectra were overlaid with a vitamin A_1_-based rhodopsin template [40].
- Ocular media transmittance
  Spectral transmittance measurements (330–800 nm) were collected from one male *C. solorensis* (*L*_S_ = 6.8 cm) euthanised with an overdose of MS222 and both eyes were immediately enucleated to avoid tissue degradation [53]. A small (approximately 2–3 mm^2^) piece of sclera was cut out of the back of each eye near the optic nerve and the underlying choroid, pigment epithelium and retina was removed to create an opening for the incident measuring beam. The light transmitted through the ocular media, lens and cornea was then measured in air [53, 54] with an Ocean Optics S2000 spectroradiometer and a xenon light source (Ocean Optics PX-2) using the setup described in detail by Theiss et al. [23]. The integration time was set to 2.5 ms with 100 scans averaged for each measurement. Three of such averaged measurements were taken per eye, resulting in a total of six averaged ocular media transmittance spectra. Each spectrum was then normalised at 700 nm and the wavelength of 0.5 transmittance (*T*_0.5_) was determined [53, 54].

### Availability of data and materials

The data set supporting the results of this article is included within the article and its Additional file 1.

## Additional file

Additional file 1: Data supplement – detailed data tables (.xls) on microspectrophotometric absorbance, ocular media transmission and fluorescence emission are provided. (XLS 226 kb)
